# Proteins in aggregates functionally impact multiple neurodegenerative disease models by forming proteasome-blocking complexes

**DOI:** 10.1111/acel.12296

**Published:** 2014-12-16

**Authors:** Srinivas Ayyadevara, Meenakshisundaram Balasubramaniam, Yuan Gao, Li-Rong Yu, Ramani Alla, Robert Shmookler Reis

**Affiliations:** 1 McClellan Veterans Medical Center, Central Arkansas Veterans Healthcare ServiceLittle Rock, AR, 72205, USA; 2 Department of Geriatrics, University of Arkansas for Medical SciencesLittle Rock, AR 72205, USA; 3 BioInformatics Program, University of Arkansas for Medical Sciences and University of Arkansas at Little RockLittle Rock, AR, 72205, USA; 4 National Center for Toxicological Research, Food & Drug AdministrationJefferson, AR, 72079, USA; 5 Department of Biochemistry & Molecular Biology, University of Arkansas for Medical SciencesLittle Rock, AR, 72205, USA

**Keywords:** Alzheimer (disease), *C. elegans*, Huntington (disease), neurodegeneration, (protein) aggregation, proteasome

## Abstract

Age-dependent neurodegenerative diseases progressively form aggregates containing both shared components (e.g., TDP-43, phosphorylated tau) and proteins specific to each disease. We investigated whether diverse neuropathies might have additional aggregation-prone proteins in common, discoverable by proteomics. *Caenorhabditis elegans* expressing *unc-54p*/Q40::YFP, a model of polyglutamine array diseases such as Huntington's, accrues aggregates in muscle 2–6 days posthatch. These foci, isolated on antibody-coupled magnetic beads, were characterized by high-resolution mass spectrometry. Three Q40::YFP-associated proteins were inferred to promote aggregation and cytotoxicity, traits reduced or delayed by their RNA interference knockdown. These RNAi treatments also retarded aggregation/cytotoxicity in Alzheimer's disease models, nematodes with muscle or pan-neuronal Aβ_1–42_ expression and behavioral phenotypes. The most abundant aggregated proteins are glutamine/asparagine-rich, favoring hydrophobic interactions with other random-coil domains. A particularly potent modulator of aggregation, CRAM-1/HYPK, contributed < 1% of protein aggregate peptides, yet its knockdown reduced Q40::YFP aggregates 72–86% (*P *< 10^−6^). In worms expressing Aβ_1–42_, knockdown of *cram-1* reduced β-amyloid 60% (*P *< 0.002) and slowed age-dependent paralysis > 30% (*P *< 10^−6^). In wild-type worms, *cram-1* knockdown reduced aggregation and extended lifespan, but impaired early reproduction. Protection against seeded aggregates requires proteasome function, implying that normal CRAM-1 levels promote aggregation by interfering with proteasomal degradation of misfolded proteins. Molecular dynamic modeling predicts spontaneous and stable interactions of CRAM-1 (or human orthologs) with ubiquitin, and we verified that CRAM-1 reduces degradation of a tagged-ubiquitin reporter. We propose that CRAM-1 exemplifies a class of primitive chaperones that are initially protective and highly beneficial for early reproduction, but ultimately impair aggregate clearance and limit longevity.

## Introduction

Many neurodegenerative diseases show age-dependent accrual of protein aggregates in affected tissues (Miller *et al*., 2010; Ratovitski *et al*., 2012), diagnostic of specific pathologies and their progression. Although large aggregates may be protective by sequestering neurotoxic soluble oligomers (Nucifora *et al*., 2012), aggregation must promote some neuropathies since heritable disease clusters often feature mutations that increase protein misfolding. Huntington's or Parkinson's pedigrees assort with mutations favoring aggregation (Lesage & Brice, 2009; Arrasate & Finkbeiner, 2012), and failure of ‘proteostasis’ (protein homeostasis) precedes neurotoxicity in many such diseases (Kikis *et al*., 2010; Liachko *et al*., 2010). Familial amyotrophic lateral sclerosis can arise from mutations affecting *SOD1* (superoxide dismutase) or *UBQLN-2* (a ubiquitin targeting damaged proteins to proteasomes), defects likely to compromise proteostasis. Alzheimer's disease (AD) features two distinct types of protein aggregates, ß-amyloid seeded by Aß_1–42_ peptide (Youmans *et al*., 2012) and neurofibrillary tangles initiated by tau aggregation (Ittner *et al*., 2010). Thus, most major neuropathies involve defects leading to damage, misfolding, and aggregation of susceptible proteins. A few proteins (tau, α-synuclein, TDP-43) are shared by aggregates for several diseases each, differing with respect to colocalizing components and the brain regions affected (Gitler *et al*., 2009; Ittner *et al*., 2010; Bigio, 2011).

Specific genetic lesions, perhaps exacerbated by exposure to toxic chemicals (Gitler *et al*., 2009), may determine the site of neuropathy as the ‘weakest link’ based on the balance of factors eliciting or opposing aggregation. Although the mechanisms are poorly understood, aggregation is thought to be favored by local abundance, modification, and structural instability of vulnerable proteins (Wright *et al*., 2005; Liachko *et al*., 2010; Dillin & Cohen, 2011) and opposed by chaperones, ubiquitin–proteasomal clearance, and autophagy (Bennett *et al*., 2007; Jia *et al*., 2007).

*Caenorhabditis elegans* models of protein aggregation have provided some key insights into mechanisms that promote aggregation (Guthrie *et al*., 2011) or oppose it (Kikis *et al*., 2010; Dillin & Cohen, 2011). Strain AM141, expressing an integrated *unc-54p/Q40::yfp* transgene in body-wall muscle, forms fluorescent intramyofibrillar foci that increase in number and brightness until at least 5–6 days posthatch (d_PH_) at 20 °C (Morley *et al*., 2002). Foci are reduced by life-extending interventions such as *age-1* mutation (Morley *et al*., 2002) and exposure to salicylate or aspirin (Ayyadevara *et al*., 2013). However, aggregation cannot be the main cause of mortality, as interventions that reduce foci may not alter lifespan (Cohen *et al*., 2010; van Ham *et al*., 2010). A variety of proteins accumulate in normal worm aggregates with age (David *et al*., 2010). Although this suggests that some aspect of aging may favor aggregation, most models of protein aggregation were never intended to reflect age-dependent processes. Some depend on induction rather than aging (Drake *et al*., 2003), while others appear independent of age during early adulthood (Christie *et al*., 2014) or respond to aging only quite early in adult life (Morley *et al*., 2002; Ben-Zvi *et al*., 2009). Because protein aggregation must reflect the molecular environment in which it occurs, assays with late-life endpoints should model the strong age dependence of human neuropathies better than those employing early induction. We therefore modified two models of AD aggregation, to delay their pathology until much later in nematode life.

A mutagenesis approach to finding modulators of protein aggregation, which also used *C. elegans* strain AM141, reported just one locus at which mutations alter aggregation: an uncharacterized gene termed *moag-4* (van Ham *et al*., 2010). Here, we present the results of a proteomics approach to identify protein components of purified Q40::YFP-containing aggregates and to assess their functional roles. For three of four tested genes encoding aggregated proteins, RNAi knockdown reduced aggregates and/or behavioral deficits in at least two *C. elegans* models of aggregation-induced cytotoxicity. Thus, proteomics in such model systems may be an efficient means to define candidate targets for functional testing by RNA interference.

## Results

### Isolation and characterization of proteins in *C. elegans* aggregates

**Figure 1:**
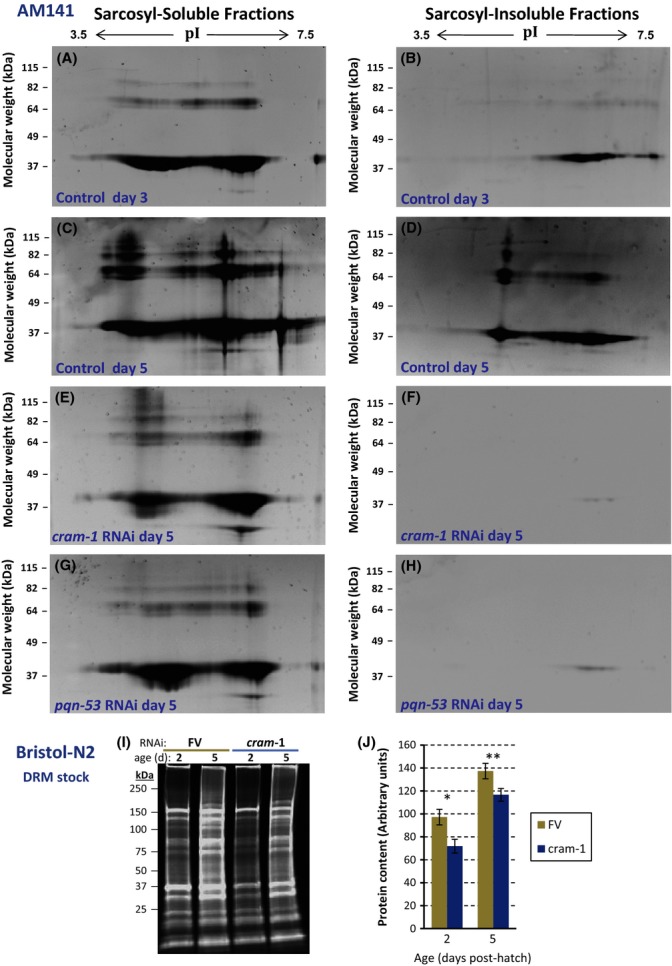
Aggregated proteins from *Caenorhabditis elegans* adults expressing Q40::YFP in muscle. (A–H): center areas of 2D gels, stained with SYPRO Ruby, resolving proteins from aggregates pulled down with antibody to GFP. Worms were grown from hatch on ‘FV’ bacteria without RNAi (A–D) or expressing dsRNA targeting *cram-1* (E, F) *or pqn-53* (G, H). Aggregates, isolated from strain AM141 at 3 d_PH_ (A, B) or 5 d_PH_ (C–H), were partitioned into those soluble (A, C, E, G) or insoluble (B, D, F, H) in 1% sarcosyl. Lanes contain equal worm equivalents of aggregated proteins, dissolved in Laemmli buffer at 95 °C. Material at ∼40 kDa binds antibody to GFP and thus may comprise modified/degraded fragments of Q40::YFP. Sarcosyl-soluble and sarcosyl-insoluble aggregate proteins from wild-type/Bristol-N2 at 2 d_PH_ (L4 larvae) or 5 d_PH_ (adults) were resolved by 1D electrophoresis (I) and quantified (J). *Two-tailed *t*-test *P *< 0.003; **P < 0.0003.

We harvested AM141 adults at several ages and isolated aggregates by immuno-affinity to magnetic beads coated with anti-GFP immunoglobulin (IgG) that also binds YFP. Beads were rinsed to remove organelles and debris, prior to release of adsorbed complexes. Recovered aggregates were partitioned by solubility at 22 °C in 1% (w/v) sarcosyl (sodium dodecyl sarcosinate), an anionic detergent less nucleophilic than sodium dodecyl sulfate (SDS). Sarcosyl-insoluble aggregates are thought to be larger, extensively cross-linked conglomerates (Liachko *et al*., 2010), but soluble complexes may be more neurotoxic (Nucifora *et al*., 2012). Figure1 shows central regions of denaturing 2D gels, displaying aggregate protein fractions dissolved at 95 °C in SDS plus β-mercaptoethanol. Both sarcosyl-soluble (panels A, C) and sarcosyl-insoluble fractions (B, D) show increases with age in the quantity and diversity of aggregated proteins. Aggregate quantity reproducibly plateaus after ∼5 d_PH_ (with two- to threefold more protein than at 3 d_PH_), while complexity (the number of constituent proteins) continues to rise for ≥ 9 days.

To identify proteins that co-aggregate with Q40::YFP, sarcosyl-soluble and sarcosyl-insoluble aggregates were isolated from AM141 worms at 4 or 7 d_PH_, digested with trypsin, and their proteins separately analyzed on an LTQ Orbitrap-FT mass spectrometer. Proteins identified with high confidence (false discovery rate *q *< 0.01) include YFP, nine prion-like proteins, six neuropeptides, three S/T-kinases, two S/T-phosphatases, six membrane-transporter subunits, two ubiquitin-related proteins, two proteasome subunits, six RNA-binding proteins, three glycoproteins, an amyloid-like protein, and an S6 (S/T/Y) kinase (see Table S1, Supporting information).

We selected proteins for functional assays, based on presence in the day 7 insoluble fraction and homology to proteins implicated in neurodegeneration. PQN-53, the most abundant of nine proteins with a glutamine/asparagine-rich (prion-like) domain, comprised 34% of insoluble protein hits (ninefold enrichment over soluble aggregates). PQN-53 interacts with SPR-2 (Zhong & Sternberg, 2006), which regulates expression of presenilin orthologs *sel-12* and *hop-1* (http://www.wormbase.org). PQN-22, the next most abundant prion-like protein, is uncharacterized. ATX-2 is orthologous to human ataxin 2, which when mutated leads to spinocerebellar ataxia-2 (SCA2); conservation is moderate over 66% of ATX-2 sequence (blastp*e *= 2E-26 to human). An uncharacterized protein, F13G3.10, was identified only in the day 7 insoluble fraction. Its closest mammalian homolog is HYPK, huntingtin-interacting protein K. Because this homology is very modest (*e *= 4E-8, conserving 34/129 amino acids), F13G3.10 may be considered a novel protein, which we term ‘cytotoxicity-related aggregation mediator-1’ (CRAM-1).

### Knockdown of *cram-1* or *pqn-53* lowers the number and protein content of Q40 aggregates

**Figure 2:**
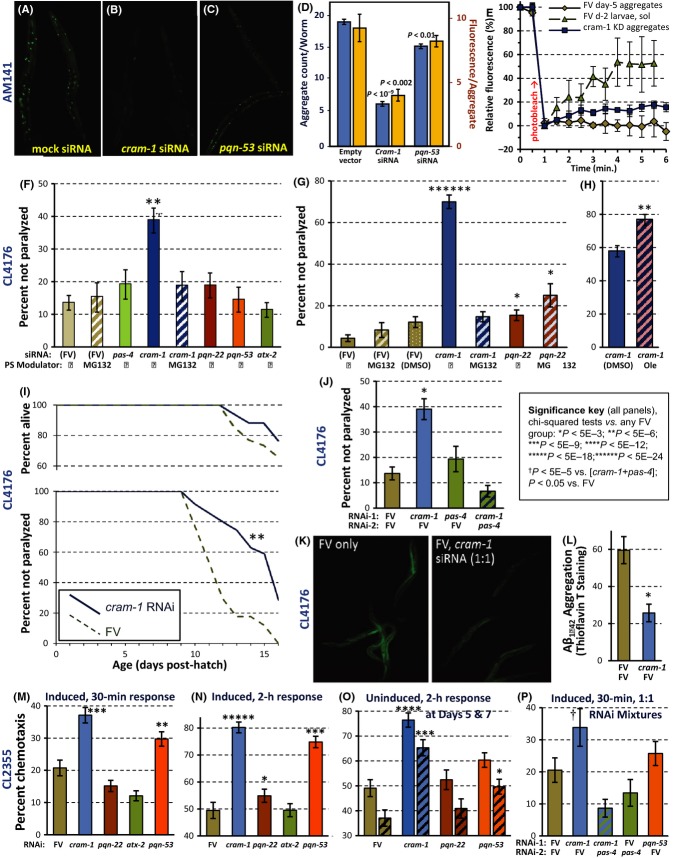
Aggregates and associated traits are lessened by RNAi. (A–D) For 3 days from the L4/adult molt, worms were fed *E. coli* with empty vector (FV) or expressing dsRNA targeting *cram-1* or *pqn-53*. Worms were imaged on 5 d_PH_ (adult day 2.5). (A–C), fluorescence images; (D) aggregate count (blue bars, left scale) and intensity/aggregate (orange bars, right scale), ± SEM, for *N *= 12–14 worms/group. Each knockdown was compared to control by two-tailed *t*-tests. (E) Fluorescence recovery after photobleaching was assessed as described (van Ham *et al*., 2009). For times ≥ 4 min, each group differed from either other group by two-tailed *t*-test, *P* < 2E-6. (F) CL4176 worms were exposed to RNAi or MG132 from the L3/L4 molt; *myo-3p*/Aß_1–42_ was induced 48 h later by upshift to 25 °C, assaying paralysis 32 h later. (G, H), paralysis 28 h postinduction, as (F) except treatments began at hatching, with Aß_1–42_ induced 48 h later. (F–H), MG132 was added at 20 μm, oleuropein (Ole) at 80 μg mL^−1^; *N *> 150/group. (I) Uninduced CL4176 (at 20 °C) undergo age-dependent paralysis, delayed ∼30% by *cram-1* RNAi fed from the L4/adult molt (*P *< 10^−6^; *N *= 34–35/group). (J) CL4176 worms, fed dual RNAi from early L4, had Aß_1–42_ induced at 48 h; paralysis and amyloid were measured 32 h later. The *cram-1*/FV RNAi mix (blue bar) reduced paralysis below other groups (each *P *< 0.001); in two repeats, each *P *< 0.05, 0.002. (K) CL4176 adults were stained with thioflavin T (ThT) after 3 days on FV or a 1:1 mixture of FV and *cram-1* RNAi. (L) ß-amyloid staining with ThT fell ∼60% with *cram-1* RNAi (*P *< 0.001, one-tailed *t-*test). (M) Impaired chemotaxis to *n*-butanol, in CL2355 worms expressing pan-neuronal Aß_1–42_. *Induced worms* (M, N, P), RNAi-treated from the L3/L4 molt, were upshifted 48 h later. Chemotaxis (%) was scored after 0.5 or 2 h. *Uninduced worms* (O) were fed RNAi from hatch. Chemotaxis declined between d5 (solid bars) and d7 (hatched bars). (P) Worms were fed dual RNAi bacteria (1:1) as indicated. (F–H, J, M–P) Error bars show standard errors of proportions. Key and legend: unadjusted chi-squared *P* values are shown, *N *= 50–200/group. Similar results were obtained in repeats for each panel.

AM141 worms were fed from the last (L4) larval stage on bacteria expressing double-stranded RNA (dsRNA) targeting the above candidate genes or carrying empty feeding vector (FV) as control. The efficiency of knockdown, assessed by RT–PCR in three experiments, was 62–94% for *cram-1* (each *P *< 0.001) and 47–98% for other genes tested (not shown). Knockdown of five genes, encoding proteins identified in Q40::YFP aggregates, resulted in significantly fewer aggregates (Table S2, Supporting information). With RNAi started just before adulthood to avoid developmental effects, Q40::YFP foci were reduced ∼50% by *cram-1* knockdown. That efficacy rose to ∼65% when RNAi was initiated at hatching (Fig.2A–D). Total punctate Q40::YFP signal in the latter experiment fell 86% with *cram-1* knockdown [i.e., 1– (0.35 × 0.39); *P *< 10^−12^], although CRAM-1 probably represented < 1% of aggregate proteins (based crudely on spectral counts). In contrast, RNAi against *pqn-53* reduced focal signal 28% [1 − (0.83 × 0.87); *P *< 0.004], despite encoding the most abundant protein in aggregates, accounting for 27% of peptide hits. This implies that CRAM-1 plays a highly ‘leveraged’ role in aggregate accrual, whereas PQN-53 promotes aggregation roughly to the extent it contributes.

*Caenorhabditis elegans* proteins shown to modulate aggregation include AGE-1 (the class-I catalytic subunit of PI3K, central to insulin-like signaling) (Morley *et al*., 2002), DAF-16 [a transcription factor (TF) mediating insulin-like and nutrient signaling] (Cohen *et al*., 2010), heat-shock response factor (HSF-1) (Cohen *et al*., 2010), and PHA-4 (a TF that mediates caloric restriction, competing with DAF-16) (Panowski *et al*., 2007). To determine whether those pathways contribute to proteostatic benefits of *cram-1* knockdown, we fed worms on 1:1 mixtures of RNAi constructs or diluted with empty vector (FV) to assess single knockdowns (Min *et al*., 2010). Our data (Table1) show that none of these pathways is needed for *cram-1* RNAi to improve proteostasis. On the contrary, all but *daf-16* show modest synergy with *cram-1* so that the benefit of combining their RNAi's is greater than expected for independent pathways (the product of gains from each alone).

**Table 1:** Dual RNAi effects on mean aggregate numbers per worm

RNAi-1	RNAi-2	Mean count	SEM	KD, % of control	Two-tailed *t*-test	Predicted KD% (ifindependent)
FV (empty vector)	FV	64.5	1.1	–	–	–
*age-1*	FV	52.5	2.2	81	1.5E−05	–
*daf-16*	FV	49.1	1.6	76	4.0E−09	–
*hsf-1*	FV	57.0	1.4	88	1.6E−04	–
*pha-4*	FV	53.8	1.2	83	1.6E−07	–
*cram-1*	FV	51.4	1.0	80	1.3E−09	–
*age-1*	*cram-1*	33.7	1.3	52	5.7E−18	0.81 × 0.80 = 65%
*daf-16*	*cram-1*	40.1	1.9	62	9.9E−13	0.76 × 0.80 = 61%
*hsf-1*	*cram-1*	40.7	1.0	63	1.1E−17	0.88 × 0.80 = 71%
*pha-4*	*cram-1*	38.9	1.6	60	1.5E−14	0.83 × 0.80 = 67%

Dual RNAi AM141 worms were assessed at 4 days posthatch (d_PH_), 14–19 fields per group with 1–4 worms per field. Aggregate counts for fields with multiple worms were divided by *N* and treated as single data points. Significance was assessed by two-tailed, homoscedastic *t*-tests, for differences between each indicated treatment group and the feeding vector control (FV/FV, no RNAi). Predicted knockdown percent for dual RNAi treatments was calculated by multiplying the percent of control for each RNAi used alone.

Fluorescence recovery after photobleaching (FRAP) of control/FV-fed aggregates indicated virtually no Q40::YFP mobility at 5 d_PH_ [recovery at ≥ 4 min (mean ± SEM) = –3.0 ± 2.5%], whereas the recovered/mobile fraction in L4 larvae averaged 52.5 ± 8.7% (Fig.2E). Aggregates in day 5 adults fed *cram-1* RNAi were intermediate, with 16.4 ± 1.4% mobility (differing from either FV group at *P *< 2E-6). This implies that aggregates are substantially more fluid after *cram-1* knockdown than in control adults, but not as diffuse as Q40::YFP in control larvae.

Protein separation on 2D gels revealed that RNA interference with either *cram-1* or *pqn-53* had its greatest effect on the detergent-insoluble fraction where they reside (Fig.1F and H), reducing protein content roughly 20-fold and 11-fold, respectively, relative to controls (Fig.1D). Fluorescence was measured in lanes loaded with equal worm equivalents, after staining protein with SYPRO Ruby. Sarcosyl-soluble fractions were also decreased, although more modestly, by dsRNA targeting *cram-1* or *pqn-53* (Fig.1, compare panels E and G–C).

Aggregation was then assessed in wild-type (Bristol-N2) worms at 2 and 5 d_PH_ by isolating their sarcosyl-insoluble aggregates. Proteins liberated from these natural aggregates, resolved on 1D gels, were stained and quantified. Aggregates did accrue in normal aging without any transgenic ‘seed protein’ (Fig.1I and J), confirming a previous report (David *et al*., 2010). Moreover, these data show that *cram-1* RNAi suppresses normal aggregation, although less profoundly (15–26%; each *P *< 0.003) than neuropathic aggregation.

### CRAM-1 knockdown reduces cytotoxicity in *C. elegans* models of Aβ aggregation

We next asked whether RNAi knockdown of proteins found in Q40::YFP aggregates can also impede aggregation in a nematode model of Alzheimer's disease expressing human Aß_1–42_ in muscle. As in previous studies of CL4176 (Dosanjh *et al*., 2010), *myo-3*-driven Aß_1–42_ was induced in 48-h larvae by upshift to 25 °C, causing paralysis within 24 h. For most experiments (Fig.2F and I–L), worms were fed dsRNA-expressing bacteria from the late L3 larval stage (40 h posthatch at 20 °C), to minimize developmental effects; RNAi then had an 8–9 h ‘headstart’ prior to upshift, plus a further 35 h before the first assay. Under those conditions, knockdown reduced *cram-1* transcripts 62% (*t*-test *P *< 0.001) and conferred ∼threefold protection from paralysis (Fig.2F; *P *< 10^−6^). No other RNAi was as protective, although *pqn-22* and *pqn-53* dsRNAs also conferred significant benefits in several experiments (see below). When RNAi began at hatching (Fig.2G–H), allowing 48-h exposure to *cram-1* dsRNA before Aß_1–42_ induction, 70% of worms were spared paralysis, 17-fold more than controls (*P *< 10^−27^). *Age-dependent paralysis*, observed in uninduced CL4176 worms well in advance of death (Fig.2I, compare upper and lower plots), presumably reflects undetected ‘leaky’ expression of Aß_1–42_ at 20 °C. Knockdown of *cram-1* then delayed paralysis 4 days or > 30% (log-rank *P *< 10^−6^).

#### Proteasome activity is essential for protective effects of CRAM-1 knockdown

To reduce aggregation and paralysis, *cram-1* knockdown requires functional proteasomes. This was first shown by treatment with MG132, a drug that inhibits proteasomes (Guo & Peng, 2013). MG132 prevented all benefit of *cram-1* RNAi, but not that of *pqn-22* knockdown (Fig.2F–G). Conversely, oleuropein (Ole), a proteasome activator that reduces Aβ_1–42_ aggregation in worm muscle (Diomede *et al*., 2013), improved protection by *cram-1* knockdown and even reduced aggregation in worms without *cram-1* knockdown (Fig.2H and Table S3, Supporting information). The strong dependence on proteasome activity, of paralysis rescue by *cram-1* RNAi, was confirmed by combining RNAi constructs against *cram-1* and *pas-4*, encoding a proteolytic α subunit of proteasomes. Neither MG132 nor *pas-4* RNAi alone increased Aβ_1–42_-induced paralysis (Fig.2F, G and J), yet addition of *pas-4* dsRNA completely blocked all benefits of *cram-1* RNAi (Fig.2J). *Cram-1* knockdown also reduced Aβ_1–42_/amyloid aggregation, visualized with thioflavin T, by > 60% (Fig.2K and L).

We next employed a neurotoxicity model, strain CL2355, in which Aβ_1–42_ is expressed and forms amyloid aggregates in neurons. The same candidate genes were assessed for RNAi effects on chemotaxis toward 1-butanol, a behavior disrupted by pan-neuronal Aß_1–42_ expression. Although neurons are relatively resistant to dsRNA entry (Calixto *et al*., 2010), results were quite consistent for neuronal (CL2355) and muscle (CL4176) expression of Aβ_1–42_. RNAi targeting *cram-1* was the most protective, followed by *pqn-53* and *pqn-22* (Fig.2M–P). Chemotaxis, like paralysis, was age dependent in uninduced worms (Fig.2O), worsening between day 5 (unhatched bars) and day 7 (hatched bars). Proteasome disruption by *pas-4* RNAi again blocked all benefits of dsRNA targeting *cram-1* (Fig.2P).

### Proteasomes normally confined within aggregates are diffuse when CRAM-1 is absent

**Figure 3:**
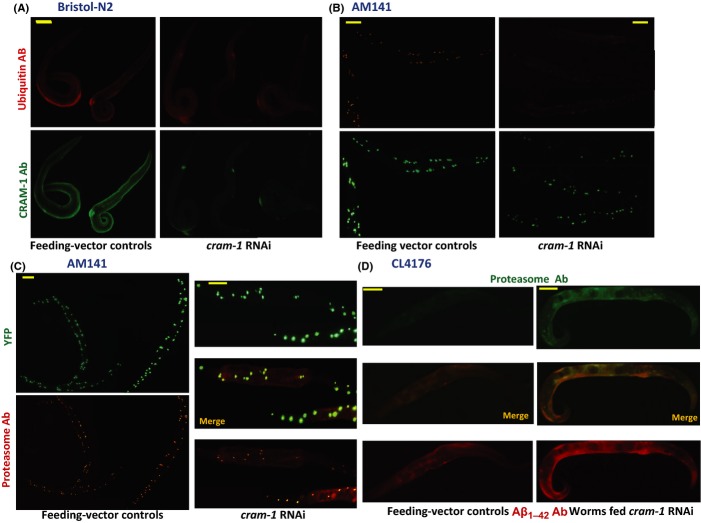
Immunofluorescence detection of *cram-1* RNAi effects on wild-type worms and worms expressing Q40::YFP or Aβ_1–42_ in muscle. (A–D) Adult *Caenorhabditis elegans*; scale bars are 0.1 mm. (A) N2 (wild-type) worms were fed *cram-1* RNAi or FV, from hatch. Adults (4 d_PH_) were immunostained with primary antibody to ubiquitin or CRAM-1. (B, C) AM141 (5 day) worms were imaged by YFP fluorescence and fluor-tagged antibody to ubiquitin (B) or proteasomes (C). (D) CL4176 worms, induced at 2 d_PH_, were imaged 40 h later with antibodies to Aβ_1–42_ and proteasomes. Channel crossover was < 5%; fluorescence was reduced > 85% in controls w/o primary antibody.

To learn how *cram-1* knockdown acts through proteasomes to alleviate aggregation, worms were immunostained *in situ* for ubiquitin or proteasomes. In N2 control worms fed bacteria harboring empty FV vector, ubiquitin (red) and CRAM-1 (green) were detected in all tissues (Fig.3A, left panels). Exposure to *cram-1* dsRNA eliminated all but traces (presumably neuronal) of signal detected by CRAM-1 antibody, while scarcely altering ubiquitin signal (right panels, Fig.3A). Q40::YFP aggregates in AM141 worms (Fig.3B and C) fell 45% in number (*t*-test *P *< 3E−5) and 49% in intensity (*P *< 0.01) after *cram-1* RNAi. Ubiquitin signal coincided with the centers of Q40::YFP foci in control worms, but appeared fainter and more diffuse after *cram-1* knockdown (Fig.3B, lower panels). Proteasome signal outside the aggregates increased >twofold (3.2- and 2.2-fold in two experiments, *P *< 0.02 and < 0.0002) after *cram-1* siRNA, largely offset by a decline within aggregates (Fig.3C). In CL4176 worms (Fig.3D), *cram-1* RNAi elicited a modest (∼28%) increase in muscle Aβ_1–42_ staining 40 h after induction (red images; *P *< 0.004), while increasing proteasome signal by ∼50% (green images; *P *< 0.008).

**Figure 4:**
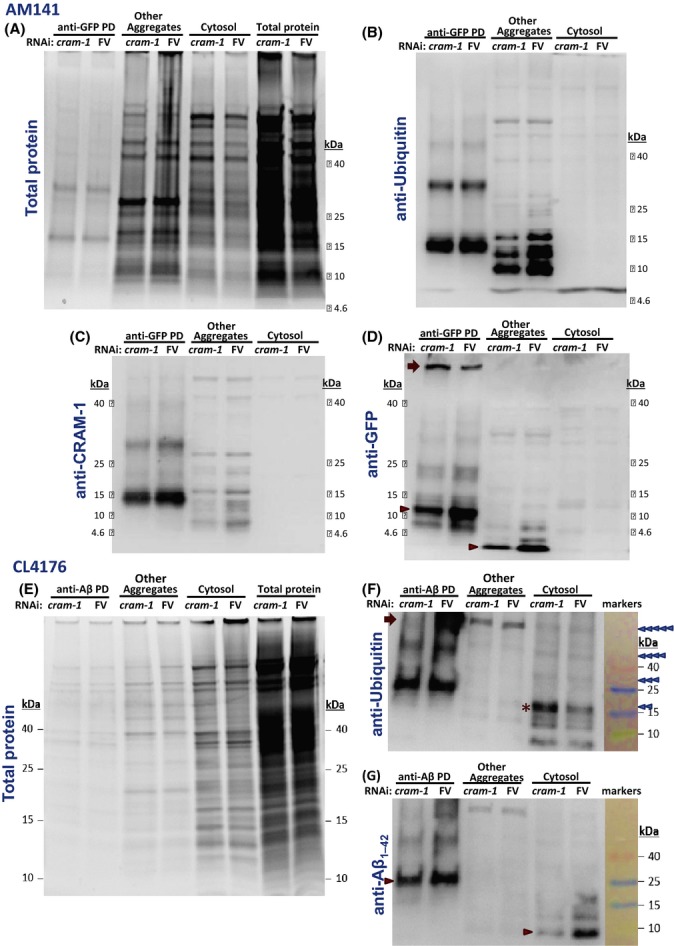
Western blot analyses of aggregated proteins from worms expressing Aβ_1–42_ or Q40::YFP. (A–D) AM141 worms, expressing Q40::YFP in muscle, were fed *cram-1* dsRNA or FV bacteria. Aggregates were isolated and fractions resolved in gradient gel lanes (8–12%, Invitrogen). Proteins were stained with SYPRO Ruby (A), or electro-blotted to nylon membranes and probed with antibodies raised to ubiquitin (B), CRAM-1 synthetic peptides (C), or GFP/YFP (D), followed by biotinylated 2nd antibody to IgG. HRP-streptavidin was bound to biotin, and imaged by chemiluminescence (Western Blot Kit, Pierce). (E–G) CL4176 worms, expressing human Aβ_1–42_ in muscle, were fed *cram-1*RNAi or FV as above. Aggregate proteins were analyzed as above. (E) SYPRO Ruby-stained proteins or fractions, separated on 4–20% gradient gels (Bio-Rad). Small proteins such as Aβ_1–42_ were resolved on 16% gels (panels F, G). After blotting, nylon filters were probed with antibodies to ubiquitin (F) or Aβ_1–42_ (G) and detected as above. Labeled size standards are shown in panels F and G. Triangles (right) indicate expected positions for di-, tri-, tetra-, and penta-ubiquitinated Aβ_1–42_.

To further characterize several constituent proteins and their modifications, aggregates and cytosol were prepared by differential centrifugation from AM141 adults at 5 d_PH_. Aggregates containing Q40::YFP were purified by pull-down (PD) on magnetic beads coated with antibody to GFP, which also binds YFP. These fractions, isolated without sarcosyl, were electrophoresed, blotted, and probed with antibodies to ubiquitin, CRAM-1, or GFP (Fig.4A–D). RNAi to *cram-1* appeared to reduce ubiquitin signal (B) about twofold, relative to total protein loaded (A), in the ‘other aggregates’ that did not bind antibody to GFP (*P*≈0.06 by two-tailed paired *t*-test), but had far less effect on GFP pull-down fractions. Signal in ‘B’ is attributed to ubiquitinylation of YFP and its degradation products, or other co-aggregated proteins, as Q40 contains no lysines to receive ubiquitin. CRAM-1 signal (C) fell ∼twofold with *cram-1* RNAi, both in the anti-GFP pull-down (*P *< 0.05) and ‘other aggregates’ (*P *< 0.02). As expected from results already shown (Figs1 and 2), RNAi targeting *cram-1* removed most of the GFP signal seen in either aggregate fraction (arrowheads, panel D, lanes 1–4; each *P *< 0.05), except for a low-mobility band that increased (arrow, panel D, lanes 1–2).

A parallel protocol was followed for the CL4176 strain at day 5, first isolating total aggregates and then separating Aβ_1–42_-containing aggregates from ‘other aggregates’ by magnetic-bead pull-down. These fractions were electrophoresed, blotted, and probed with antibodies to ubiquitin or Aβ_1–42_ (Fig.4F–G). Total protein staining (panel E) confirmed similar loads in each fraction, from worms on *cram-1* RNAi vs. vector (FV). Knockdown of *cram-1* had opposite and nonsignificant effects on ubiquitin signal in Aβ-PD aggregates [decreased at higher molecular weight (arrow)] vs. cytosol [increased, panel F (*)]. Bands at * and above may correspond to successive ubiquitin additions to Aβ_1–42_ (triangles at right). A similar ladder was also seen in a separate gel and Western blot detecting Aβ_1–42_ (G). As expected, signal was reduced by *cram-1* knockdown, both in Aβ-PD aggregates (33%, *P *< 0.02) and in cytosol (82%, *P *< 0.03), especially at bands marked by arrowheads (G).

Consistent with our *in situ* staining (Fig.3) and previous reports (Hallengren *et al*., 2013), aggregates in control worms had > 3 times more ubiquitinylation (*P *< 0.03) than equal worm equivalents of unaggregated proteins, although this difference largely disappeared with *cram-1* RNAi [Fig.4B and F; in each panel, compare aggregates (lanes 1–4) to cytosol (lanes 5, 6)].

### CRAM-1 can interact with ubiquitin and its oligomers to form tightly condensed complexes

**Figure 5:**
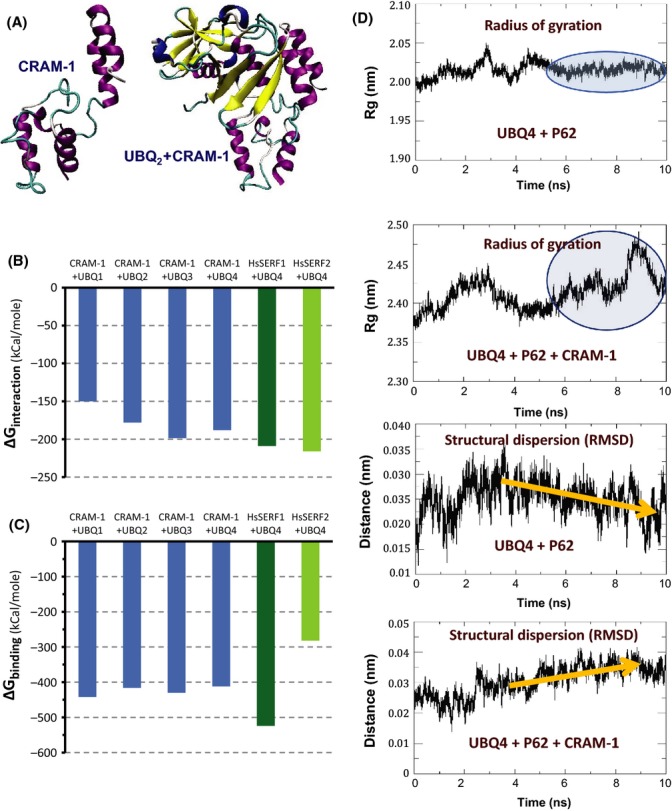
Molecular dynamic modeling of CRAM-1 interactions with other proteins. (A) Ribbon structure model of CRAM-1 alone or interacting with di-ubiquitin created with modeller 9.12 and viewed with vmd software. Interaction energy change ΔE (B) and binding energy change ΔG (C) were calculated from molecular dynamic simulations of ≥ 10 ns, under gromacs, for mono-, di-, tri-, and tetra-ubiquitin interacting with CRAM-1 (blue bars) or its human orthologs SERF1 and 2 (green bars). (D) (Top panels) Radius of gyration (Rg) calculated for tetra-ubiquitin (UBQ4) interacting with p62 (sequestosome-1) or with p62+CRAM-1. Blue ovals show regions of stable behavior for the first simulation, which becomes chaotic when CRAM-1 is added. (Lower panels) Root-mean-square distance between molecular centers of mass (RMSD, an index of structural dispersion), for UBQ4 interacting with p62, or p62+CRAM-1. Gold arrows show intermolecular distance narrowing for UBQ4+p62 interaction, but increasing for UBQ4+p62+CRAM-1.

We next characterized known and predicted features of CRAM-1. CRAM-1 lacks any defined protein motifs (Prosite.expasy.org) and is ≥ 95% disordered (Schlessinger *et al.,* 2009). It is a highly charged protein, with a marked acidic bias (21 acidic and 14 basic residues of 96). Molecular dynamic simulations (gromacs) show CRAM-1 alone adopting > 80% random-coil conformation, whereas it forms compact complexes with ubiquitin or oligomers thereof (Fig.** **5A). These complexes have negative energies of interaction (Fig.5B, CRAM-1 bars), implying that the constituents would interact spontaneously, and are quite stable with ΔG's of 400–530 kCal mole^−1^ below the sum of constituents evaluated separately (Fig.5C, CRAM-1 bars). Human orthologs of CRAM-1, related by descent but so diverged as to lack any significant homology to it (*e* ≥ 1), show quite similar interaction and stabilizing free-energy changes (Fig.5B and C, SERF1 and 2). By way of comparison, ΔG and ΔE values as small as 20–30 kCal mole^−1^ are sufficient to allow spontaneous interaction and to confer stability (Singam *et al*., 2013).

Are there predicted functional consequences of this interaction, for accessibility of polyubiquitin to trigger proteasomal degradation or autophagy? Although it is not currently possible to model the interaction of ubiquitin ± CRAM-1 with the proteasome ‘cap’ structure, we instead modeled the well-defined interaction of ubiquitin ± CRAM-1 with ‘sequestosome-1’ (SQST-1/p62) that mediates docking of polyubiquitinylated proteins to proteasomes and autophagosomes (Jia *et al*., 2007). Although the complex of p62 with ubiquitin stabilizes over time (indicated by flattening of the radius of gyration (top panel, Fig.5D) and decreasing distance between the molecular centers of mass (3rd panel, Fig.5D), the addition of CRAM-1 is predicted to destabilize the resulting ternary complex (implied by high variability in the radius of gyration and increasing separation between the centers of mass (2nd and 4th panels, Fig.5D). This suggests an intriguing and novel role for CRAM-1, opposing the targeting function of ubiquitinylation.

### RNAi targeting *cram-1* impairs proteasomal degradation

**Figure 6:**
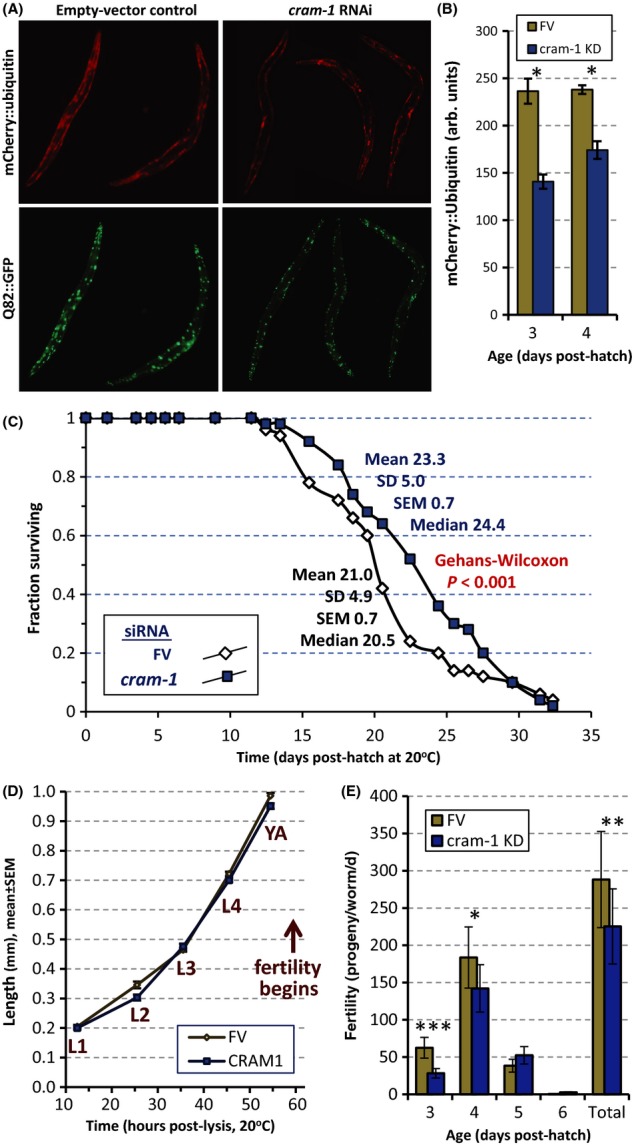
RNAi to *cram-1* extends lifespan and lowers fecundity. (A) Images of worms expressing mCherry::ubiquitin (upper panels) and Q82::GFP (lower) in body wall muscle, at 3 d_PH_ (adult day 1). Worms were fed FV bacteria (left panels), or *cram-1* siRNA bacteria (right panels). (B) Red fluorescence (mCherry-ubiquitin) was quantitated by ImageJ (http://fiji.sc/Fiji), from images as in A, 10–12 worms per group, at 3–4 d_PH_ (adult days 1–2). *Each two-tailed *t-*test *P *< 6E-6. The mean decline in fluorescence, at 3 d_PH_ (two repeats), was 46%. (C) Lifespan data for wild-type *Caenorhabditis elegans* fed from the L4/adult molt on FV or *cram-1*-dsRNA bacteria. Worms were transferred to fresh plates daily for 8 day to remove progeny and scored at 1- or 2-day intervals for movement (spontaneous or after gentle prodding). In two independent experiments (35 worms/group), *cram-1* RNAi extended mean lifespan 11–12% (each *P *< 0.001, Gehans–Wilcoxon test). (D) Worm dimensions were measured at the indicated stages (larval, L1–L4; young adult, YA) and times (*x*-axis, hours after egg isolation), using WormSizer, a Fiji plug-in (http://fiji.sc/Fiji). Lengths (shown) and widths (not shown) did not differ significantly at any time, between worms receiving *cram-1* dsRNA or FV control bacteria, in three experiments. Error bars, often smaller than symbols, show ± SEM. (E) Fertility data for worms maintained as in (D). Parents were moved to fresh plates at 24-h intervals; L2 larvae were counted 24 h later for 10–14 plates per group. Similar results were obtained in replicate experiments. Significance by two-tailed *t*-tests, *cram-1* KD vs. FV control: *P < 2E-3; **P < 4E-4; ***P < 2E-5.

The prediction based on molecular modeling that CRAM-1 will interact with ubiquitin to disrupt its interactions with proteasomes and autophagosomes was tested *in vivo* using a reporter strain to monitor degradation of an artificial proteasome substrate in body wall muscle. In this construct, *unc54p*/mCherry::ubiquitin is initially diffuse but is partially recruited to Q82::GFP aggregates during the L4 larval stage (Skibinski & Boyd, 2012). As shown in Fig.** **6A and B, adult worms on empty vector have numerous Q82::GFP aggregates and relatively high mCherry::ubiquitin levels, much of which colocalizes with Q82::GFP. This reporter for undegraded ubiquitin was significantly reduced by *cram-1* knockdown (nearly twofold at 3 d_PH_, in two experiments; each *P *< 6E-6), confirming that normal CRAM-1 levels restrict proteasomal activity.

### Knockdown of *cram-1* extends nematode lifespan but reduces early fertility

To evaluate the life-history impacts of reduced *cram-1* expression, we first compared the lifespans of worms transferred at the L4/adult molt (∼2.5 d_PH_) to bacteria expressing dsRNA against *cram-1* vs. control bacteria. As shown in Fig.6C (one of two very similar replicates), *cram-1* RNAi extended mean adult lifespan by 11%, and the median by 19% (*P *< 0.001). This confirms a similar lifespan extension reported previously, in a study that listed the F13G3.10 protein (based on its homology to HYPK) as a putative component of a protein interaction network affecting longevity (Bell *et al*., 2009).

Developmental rate, indicated by measuring the length of worms as they progress through the larval stages, was unaffected by *cram-1* knockdown (Fig.6D). Small intergroup differences at L2 and young adult (YA) stages were not significant (*P *= 0.38) and were not reproduced in replicate experiments. By contrast, this knockdown reduced early fertility markedly and significantly (Fig.6E): by 55% (*P *< 2E-5) on day 3 and 23% (*P *< 2E-3) on day 4. Total progeny fell 22% with *cram-1* RNAi (*P *< 4E-4), which over ten generations would reduce the population 12-fold if food is not limiting. However, the effect on *early reproduction* is far more powerful, potentially leading to a 288 000-fold deficit over 100 days. Thus, the CRAM-1 protein enhances reproduction, and in particular *early* reproduction, traits strongly favored by natural selection, despite promoting aggregate formation and restricting longevity.

## Discussion

Proteomic analysis of toxic aggregates, formed in a simple heterologous model (*C. elegans*), identified proteins that contribute structurally and functionally to aggregates induced by quite distinct seed proteins. Nine of the endogenous proteins identified, comprising ∼33% of aggregate ‘hits’, contain unstructured (Gln/Asn-rich ‘pQN’) prion-like domains able to engage Q40 via hydrophobic interactions (Vitalis *et al*., 2008). The component most influential for aggregation, and most detrimental to muscle and neurons, was a novel protein we call CRAM-1. Its closest human homolog, HYPK, conserves 28% identity with CRAM-1, but their knockdowns have opposite effects: RNAi to HYPK increases huntingtin::eGFP aggregation (Arnesen *et al*., 2010), whereas *cram-1* RNAi reduces aggregates (Figs3). HYPK disrupts aggregation both via chaperone-like interactions and by cooperating with the NatA complex in protein N-terminal acetylation (Arneson *et al.,* 2010), while CRAM-1 promotes aggregation and associated phenotypes by blocking ubiquitin–proteasome activity, a mechanism thought to have been excluded for HYPK (Arnesen *et al*., 2010).

### Western blots and *in situ* immunostaining assess different protein states

Evidence from protein gels (Fig.1) was for the most part consistent with aggregate quantitation in worms (Figs2 and 3) and with aggregation-dependent behavioral parameters (motility, paralysis, and chemotaxis, Fig.2). However, quantitative differences between these measures were observed reproducibly and are likely to reflect real differences in the state of the molecules detected. For example, proteins ‘buried’ inside aggregates may be less accessible to antibodies and underreported *in situ*, but correctly quantitated on Western blots. Conversely, proteins in large, insoluble aggregates that are well detected *in situ* may not fully disaggregate upon heating in Laemmli buffer (especially if cross-linked), may fail to fully enter a gel, and would transfer poorly if at all to membranes for subsequent detection.

### Role of proteasomes

Proteasome dysfunction has been implicated previously in protein aggregation (e.g., Holmberg *et al*., 2004), but little is known about the nature of proteasome–aggregate interactions. When cultured cells or transgenic mice express huntingtin with expanded polyglutamine tracts, most proteasomes colocalize with aggregates in cytoplasmic and nuclear foci (Holmberg *et al*., 2004). Interventions that increase ubiquitinylation or activate proteasomes provide partial relief from aggregation-associated neurotoxicity (Diomede *et al*., 2013). Colocalization of proteasomes with nematode polyQ and Aβ aggregates (Fig.3C and D) confirms and extends previous reports (Holmberg *et al*., 2004); moreover, the liberation (diffuse localization) of proteasomes and reduced aggregate load after *cram-1* RNAi argues that proteasomes are disabled by CRAM-1 complexes in an aggregate-embedded state.

Molecular dynamic simulations and energetic analyses suggested a direct role for CRAM-1 in disrupting proteostatic clearance of misfolded and aggregated proteins, by forming stable complexes with (poly)ubiqui-tin modifications that target dysfunctional proteins for degradation. That prediction was supported by experimental data showing less degradation of a tagged-ubiquitin reporter in the presence of CRAM-1 than after its knockdown (Fig.6A and B). The present observations, and those in a previous report (van Ham *et al*., 2010), can now be interpreted in a single mechanistic context: Disordered proteins with a high charged-residue content and extreme pI may be favored by natural selection without conservation of sequence motifs or domains, based only on their ability to form stable electrostatic and hydrophobic interactions with subsets of misfolded proteins or directly with ubiquitin, condensing their structures and limiting further interactions. Such complexes, if resistant to digestion, may also be noncompetitive inhibitors of proteasomes.

### Gene ancestry and inference of protein functions

We sought mammalian orthologs of CRAM-1, based on hierarchical identification of sequences most conserved between species with a common ancestor; TreeFam (www.treefam.org) identified HYPK as the most likely human ortholog of CRAM1, whereas Inparanoid7 (inparanoid.sbc.su.se) identified SERF2 (small EDRK-rich factor-2). Figure S1A (Supporting information) shows the maximum-likelihood phylogenetic tree from TreeFam. HYPK is of great interest due to its prior association with Huntington's disease, where it appears to play a protective role (Arnesen *et al*., 2010), whereas SERF2 is predicted to promote aggregation, like CRAM-1, offering a better target for intervention.

*Caenorhabditis elegans* contains another ortholog of human SERF1/SERF2 (Fig. S1B, Supporting information), discovered by a mutagenesis screen for nematode modulators of Q40::YFP aggregation (van Ham *et al*., 2010). This protein, MOAG-4, is functionally analogous to CRAM-1 in that *moag-4* loss-of-function mutation decreased Q40::YFP aggregation ≥ 60% and reduced aggregation and paralysis in strains expressing human Aβ or α-synuclein (van Ham *et al*., 2010). Like CRAM-1, MOAG-4 was chiefly associated with detergent-insoluble aggregates (van Ham *et al*., 2010).

However, MOAG-4 differs in many respects from CRAM-1. MOAG-4, an 82-amino acid protein of predicted isoelectric point (pI) 11.0, matches 32 of 44 N-terminal residues in human SERF1 (170 amino acids, pI 11.2), and 25 residues in SERF2. CRAM-1 (96 amino acids, pI 4.4), by contrast, retains no significant homology to mammalian SERF proteins. These pI values are notably extreme: < 1% of *C. elegans* proteins have pI ≥ 11 and < 2% have pI ≤ 4.4 (Kiraga *et al*., 2007). CRAM-1 has 35 charged amino acids of 96 (37%) with a net charge of −7, whereas MOAG-4 has 31 charged residues of 82 (38%) with a +9 net charge. Although highly disordered and devoid of known protein motifs, both are capable of extensive hydrophobic and electrostatic interaction with appropriate target proteins (hydrophobic and either basic or acidic, respectively). Knockdown of *cram-1* extends nematode lifespan 11–19% (Fig.6A; Bell *et al*., 2009), whereas a *moag-4* loss-of-function mutant *reduced* survival 8–11% (van Ham *et al*., 2010). Proteasomes and autophagy were considered to be excluded as mediators of MOAG-4 (van Ham *et al*., 2010), whereas *cram-1* knockdown requires proteasome activity to oppose aggregation (Fig.2) and improves proteasomal degradation (Fig.6B), implying that proteasomes mediate CRAM-1 blockage of aggregate formation.

To reconcile these observations, we propose that the ancestral *SERF* gene underwent successive duplications to create *SERF1* (which reduplicated recently, creating *SERF1A* and *SERF1B*), *SERF2*, and a partial copy encoding HYPK. In mammals, *SERF2* and *HYPK* diverged in sequence while preserving tight genetic linkage, likely due to cotranscription (see Fig. S1C, Supporting information). The SERF2 ortholog in nematodes, CRAM-1, is so diverged from SERF proteins as to retain no reliable homology. Both CRAM-1 and MOAG-4 appear to have evolved under selection for ability to condense unstructured or unfolded proteins, but not for conservation of any protein motifs. Although beneficial early in life, and favoring early reproduction, this crude mechanism of sequestration eventually creates an unsustainable aggregate burden.

### CRAM-1 impedes the clearance of misfolded proteins targeted for degradation

Previous studies showed that polyQ tracts are incompletely digested by eukaryotic proteasomes, which then become sequestered in aggregates (Holmberg *et al*., 2004), but no mechanisms were implicated. Our results are consistent with a model in which CRAM-1, itself an inherently disordered protein, coalesces with other disordered or misfolded proteins, and in particular with their polyubiquitin tags. It then obstructs proteasomal removal of ubiquitin adducts and digestion of the tagged proteins, as well as targeting of larger aggregates to autophagy, via binding to sequestosome-1/p62. Knockdown of *cram-1* frees proteasomes from entrapment, enabling them to digest proteins in (or destined for) large aggregates, while also relieving blockage of autophagy.

CRAM-1 and MOAG-4 may represent an ancient class of primitive chaperones that evolved to interact stably with different sets of misfolded proteins, and in the case of CRAM-1, with ubiquitin tags. Although CRAM-1 impairs survival (Fig.6A), from an evolutionary perspective, this is massively outweighed by increased early reproduction in its presence (Fig.6C), accounting for its widespread occurrence in diverse Caenorhabditis species.

## Experimental procedures

### Strains

Wild-type Bristol N2, subline DRM [the longest-lived of six tested N2 stocks (Gems & Riddle, 2000)], AM141 (*unc-54*/Q40::YFP), CL4176 [*smg-1*^ts^ (*myo-3*/Aβ_1–42_/long 3′-untranslated region (UTR)], and CL2355 [*smg-1*^ts^ (*snb-1*/Aβ_1–42_/long 3′-UTR)] were obtained from the *Caenorhabditis* Genetics Center (CGC). Strain LN149 (*unc-54*/Q82::GFP; *unc-54*/mCherry::ubiquitin) was kindly provided by Drs. Lynn Boyd and Gregory Skibinski (UA Huntsville, AL, USA).

### Strain maintenance and RNAi treatments

All strains were maintained on solid nematode growth medium (NGM) overlaid with *E. coli* (OP50), at 20 °C except for upshift of strains CL4176 and CL2355 to 25 °C during the L3–L4 transition to induce Aβ_1–42_. For knockdowns, well-fed worms were lysed on day 3 posthatch (day 1 as adults) to release eggs, which hatched on plates seeded with *E. coli* (strain HT115) expressing the indicated RNAi constructs (Kamath & Ahringer, 2003).

### Paralysis assay

The CL4176 strain was synchronized by lysis and transfer of unlaid eggs onto 60-mm culture plates seeded with bacteria containing control plasmid (empty vector, FV) or plasmids expressing dsRNA against targeted genes. For dual exposure to two dsRNAs, bacteria were mixed 1:1 to combine (FV+*cram-1*)*,* (FV+*pas-4*)*,* or (*cram-1*+*pas-4*). Larvae were transferred to indicated RNAi mixtures at late L4 because *pas-4* RNAi blocks development (www.WormBase.org). Worms with induced expression of Aβ_1–42_ were upshifted (20→25 °C) at the L3–L4 transition, and paralysis scored periodically from 18 h postupshift until < 40% of FV worms were motile. Uninduced worms were kept at 20 °C to assess age-dependent paralysis. To prepare synchronized cohorts for survivals, 2-μm FUdR was present in medium from before egg laying (2.5 d_PH_) until its cessation (6–7 days later) (Van Raamsdonk & Hekimi, 2011). Paralysis (loss of motility in response to touch) was scored daily from 6 d_PH_. Each experiment was performed ≥ 3 times.

### Chemotaxis assay

Chemotaxis was assessed in strain CL2355 expressing pan-neuronal Aβ_1–42_. Synchronized eggs were fed from hatch on FV control bacteria or exposed to RNAi against indicated genes. For dual dsRNA exposure (Min *et al*., 2010), 1:1 mixtures of bacteria were used: [FV+*cram-1*]*,* [FV+*pas-4*]*,* [*cram-1*+*pas-4*]*,* or [*atx-2*+*pas-4*]. Larvae were transferred to RNAi bacteria at late L4 because *pas-4* and *atx-2* dsRNA disrupt development (www.WormBase.org). Aβ_1–42_ expression was induced by upshift to 25 °C as above, or worms were maintained at 20 °C to follow the aging decline in chemotaxis. Worms from 5 d_PH_ were washed free of bacteria (3–4× in S buffer), and 50–100/assay were placed at centers of 100-mm culture plates spotted at one edge with ∼5 μL *n*-butanol as chemo-attractant (Dosanjh *et al*., 2010) plus 0.34% (w/v) sodium azide to immobilize them, and S buffer plus 0.34% azide at the opposite edge as control. Assay plates were held at 20 °C, and chemotaxis scored every 30 min. Each experiment was performed ≥ 3 times. The ‘Chemotaxis Index’ (CI) (Dosanjh *et al*., 2010) = [(worm# near attractant) − (worm# near control)]/(total worms/plate).

### Lifespan assay

Well-fed N2 (Bristol) worms were synchronized as above and transferred at the L4/adult molt to bacteria with empty vector (FV), or expressing *cram-1, pas-4,* or *atx-2* dsRNAs as described. Worms, maintained at 20 °C, were scored daily for touch response. Significance of differences between survivals was determined by Gehans–Wilcoxon log-rank tests.

### Visualization of aggregate reporters

Q40::YFP aggregates were scored for number and intensity after imaging (Olympus BX51 fluorescence microscope with DP71 camera, Center Valley, PA, USA). Incident light was filtered to 490 ± 20 nm, and emitted light was filtered to 535 ± 30 nm. Aβ_1–42_ aggregates were stained in fixed, permeabilized worms with thioflavin T (0.1% w/v), and imaged with incident light at 360–475 nm and emission at 527 ± 20 nm. Results were confirmed with a strain expressing Aβ_1–42_::GFP in muscle, observed in 470 ± 20 nm incident light, and emitted at 514 ± 20 nm.

### Fluorescence recovery after photobleaching

Fluorescence recovery after photobleaching was assessed as described (van Ham *et al*., 2010), with dual normalization to set pre-photo-bleaching fluorescence to 100% at *t*_0_, and to correct for background. Time courses depict means ± SEM for five aggregates in each of five worms. The mobile fraction was estimated as the plateau recovery value (mean of RFI values at *t *≥ 4 min, minus RFI at *t* = 1 min, just after photo-bleaching)/(RFI at *t*_0_, minus RFI at *t* = 1 min).

### Immunostaining *in situ*

Synchronized worms were rinsed, fixed and permeabilized (Bharill *et al*., 2013), then blocked 2 h with 0.2% (w/v) BSA in 50 mm phosphate buffer, pH7.6, and stained 14 h at 4 °C with primary antibodies raised in rabbit against proteasome 19S/S5A (Abcam, Cambridge, MA, USA) or CRAM-1 peptides (Genescript, Piscataway, NJ, USA)]; or in mice against ubiquitin or Aβ_1–42_ (Abcam)], diluted 1:500 in buffer with 2% (w/v) BSA (AB/2%). Worms were washed in buffer containing 0.2% BSA (‘AB/0.2%’, 5 × 30 min) and stained 2 h at 22 °C with secondary antibody: ALEXA594-labeled donkey antibody to mouse IgG (Jackson ImmunoResearch, red, West Grove, PA, USA), FITC-labeled donkey antibody to rabbit IgG (Sigma, green, St. Louis, MO, USA), or Alexa350-labeled goat antibody to rabbit IgG (Molecular Probes, blue, Grand Island, NY, USA), each diluted 1:1000 in AB/2%. After five 30-min washes in AB/0.2%, worms were slide-mounted and imaged as above.

### Affinity purification of aggregates and protein fractionation

Age-synchronized worm were pelleted, drained, and flash-frozen in liquid nitrogen. Pellets were pulverized in a mortar over dry ice and suspended in lysis buffer (20 mm Hepes pH 7.4, 300 mm NaCl, 2 mm MgCl_2_, 1% NP40, and protease/phosphatase inhibitors; CalBiochem, Billerica, MA, USA) (Morley *et al*., 2002). After centrifugation (5 min, 2000 *g*) to remove debris and particulates, protein concentration was assayed (Bradford; Bio-Rad, Hercules, CA, USA). Q40::YFP aggregates were retained by antibody to GFP (Abcam) attached to DYNAL Protein-G magnetic beads, recovered, and suspended in 0.1 m HEPES buffer with 1% sarcosyl (v/v), 5 mm EDTA, and protease inhibitors, and then centrifuged 30 min at 100 000 *g*. Equal worm equivalents of insoluble (pellet) and soluble (supernatant) fractions were suspended in 125 μL IEF loading buffer (8 m urea, 2% CHAPS, 40 mm DTT, and 0.2% Biolyte) for 2D separation (IEF, pH 3–10, followed by electrophoresis in 1% SDS, 4–12% polyacrylamide gradient gels; Invitrogen, Grand Island, NY, USA). Proteins, stained with SYPRO Ruby (Invitrogen), were visualized in a Bio-Rad Digital Imager and quantitated with quantity one software (Bio-Rad).

### Protein identification

Protein components of aggregate fractions were dissolved in Laemmli buffer containing 2% w/v SDS and 0.5% v/v ß-mercaptoethanol and heated 5 min at 95 °C. Proteins, resolved on 1% SDS acrylamide gels, were stained with SYPRO Ruby (Invitrogen) or Coomassie blue, and 1-mm slices were excised and incubated with trypsin. Peptides were analyzed by LC-MS^2^, and proteins that were identified directly with mascot or by *de novo* sequencing (Zybailov *et al*., 2009) with > 95% confidence are listed in Table1.

### Western blot detection of ubiquitinylated proteins, Aβ_1–42_, CRAM-1, and Q40::YFP

AM141 and CL4176 worms were fed bacteria carrying FV or expressing *cram-1* dsRNA. Aggregates were prepared as above through the 2000 *g* centrifugation, but were then pelleted (50 000 *g* for 15 min), resuspended, and isolated by affinity to antibody-coated magnetic beads. Aggregates and cytosol were dissolved in 1× Laemmli buffer at 95 °C, and equal worm equivalents loaded on a gradient gel (4–12% acrylamide), electrophoresed, transferred to nylon membranes, and incubated with murine antibodies to ubiquitin, Aβ_1–42_, or GFP. After incubation with HRP-coupled antibody to mouse IgG, membranes were imaged (Bio-Rad), then stripped, and reprobed with a different primary antibody to confirm mobility shifts or coincidence seen in independent runs.

### Structure generation

Amino acid sequences of CRAM-1, MOAG-4, and UBQ (*C. elegans*); and SERF-1 and SERF-2 (*H. sapiens*) were retrieved from WormBase and UniProt databases. To identify templates for homology modeling, blastp searches were run with NCBI default parameters and 3D structures were built from Protein Data Bank templates (modeller 9.12). Because identities to templates were < 50% for CRAM-1, MOAG-4, SERF-1, and SERF-2, initial structures were derived by sequential loop refinements in modeller 9.12. Energy minimization of final lowest energy target protein conformers was run fully solvated in gromacs. Stereochemical properties of modeled structures were analyzed by procheck and verfiy3d (Sharad *et al*., 2014).

### Protein–protein docking

hex 6.1 Softonic International SA, New York, NY, USA was used to perform protein–protein docking with program default parameters. Of 2000 possible clusters ranked by interaction free energies (ΔE_int_), the lowest energy conformers were taken for further analysis.

### Molecular dynamics (MD) simulations

Simulations were run in gromacs for fully solvated molecules, individually and as interacting (‘docked’) pairs. Molecular species were CRAM-1, MOAG-4, SERF-1, SERF-2, and UBQ (as mono-, di-, tetra-, and penta-ubiquitin); pairwise interactions were UBQ_1_+CRAM-1, UBQ_2_+CRAM-1, UBQ_4_+CRAM-1, UBQ_5_+CRAM-1, UBQ_4_+MOAG-4, UBQ_4_+SERF1, and UBQ_4_+SERF2. Initial structures were immersed in a 3D ‘solvent box’, 0.3–0.8 nm per side, as required for each complex. Electrostatic energies were calculated by the particle mesh Ewald method; Coulomb and van der Waals interactions were set to 1.0; and an AMBER99SB-ILDN force field was employed. Na^+^ and Cl^−^ were included as counterions to neutralize local charges. All simulations used the same parameters unless otherwise noted. Energy minimization used the steepest descent method for 5000 steps. The system was pre-equilibrated (∼100 ps) at 300°K and constant pressure, followed by 10-ns MD runs on HPC machines. Trajectories were analyzed in gromacs and viewed with VMD (visual molecular dynamics).

### Free-energy calculations

Gibbs free energies (ΔG) of individual molecules and intermolecular complexes were calculated in gromacs. The above protocol was amended so that energy minimization entailed two stages of 5000 steps each, with and without constraints. For all complexes, MD runs comprised 20 λ points (0.0, 0.05, 0.15, 0.2, … 1.0), each spanning 200–500 ps, totaling 4–10 ns. Results were analyzed with gromacs modules.

ΔG _(binding free energy)_ for the complexes was calculated using:

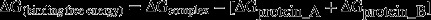
 (Singam *et al*., 2013)

## Supporting Information

**Fig. S1**SERF2-HYPK, a partially duplicated gene, is the closest human ortholog of CRAM-1.

**Table S1**Proteins identified with > 95% confidence in Q40::YFP aggregates. **Table S2** Aggregate count is reduced by RNAi targeting proteins that co-aggregate with Q40::YFP. **Table S3** Oleuropein treatment is additive with *cram-1* RNAi in suppressing Q40::YFP aggregation.

## References

[b1] Arnesen T, Starheim KK, Van DP, Evjenth R, Dinh H, Betts MJ, Ryningen A, Vandekerckhove J, Gevaert K, Anderson D (2010). The chaperone-like protein HYPK acts together with NatA in cotranslational N-terminal acetylation and prevention of Huntingtin aggregation. Mol. Cell. Biol.

[b2] Arrasate M, Finkbeiner S (2012). Protein aggregates in Huntington's disease. Exp. Neurol.

[b3] Ayyadevara S, Bharill P, Dandapat A, Hu CP, Khaidakov M, Mitra S, Shmookler Reis RJ, Mehta JL (2013). Aspirin inhibits oxidant stress, reduces aging-associated functional declines and extends lifespan of *Caenorhabditis elegans*. Antioxid. Redox Signal.

[b4] Bell R, Hubbard A, Chettier R, Chen D, Miller JP, Kapahi P, Tarnopolsky M, Sahasrabuhde S, Melov S, Hughes RE (2009). A human protein interaction network shows conservation of aging processes between human and invertebrate species. PLoS Genet.

[b5] Bennett EJ, Shaler TA, Woodman B, Ryu KY, Zaitseva TS, Becker CH, Bates GP, Schulman H, Kopito RR (2007). Global changes to the ubiquitin system in Huntington's disease. Nature.

[b6] Ben-Zvi A, Miller EA, Morimoto RI (2009). Collapse of proteostasis represents an early molecular event in *Caenorhabditis elegans* aging. Proc. Natl Acad. Sci. USA.

[b7] Bharill P, Ayyadevara S, Alla R, Shmookler ReisRJ (2013). Extreme depletion of PIP_3_ accompanies the increased life span and stress tolerance of PI3K-null *C. elegans* mutants. Front. Genet.

[b8] Bigio EH (2011). TDP-43 variants of frontotemporal lobar degeneration. J. Mol. Neurosci.

[b9] Calixto A, Chelur D, Topalidou I, Chen X, Chalfie M (2010). Enhanced neuronal RNAi in *C. elegans* using SID-1. Nat. Methods.

[b10] Christie NT, Lee AL, Fay HG, Gray AA, Kikis EA (2014). Novel polyglutamine model uncouples proteotoxicity from aging. PLoS One.

[b11] Cohen E, Du D, Joyce D, Kapernick EA, Volovik Y, Kelly JW, Dillin A (2010). Temporal requirements of insulin/IGF-1 signaling for proteotoxicity protection. Aging Cell.

[b12] David DC, Ollikainen N, Trinidad JC, Cary MP, Burlingame AL, Kenyon C (2010). Widespread protein aggregation as an inherent part of aging in *C. elegans*. PLoS Biol.

[b13] Dillin A, Cohen E (2011). Ageing and protein aggregation-mediated disorders: from invertebrates to mammals. Philos. Trans. R. Soc. Lond., B, Biol. Sci.

[b14] Diomede L, Rigacci S, Romeo M, Stefani M, Salmona M (2013). Oleuropein aglycone protects transgenic *C. elegans* strains expressing Abeta42 by reducing plaque load and motor deficit. PLoS One.

[b15] Dosanjh LE, Brown MK, Rao G, Link CD, Luo Y (2010). Behavioral phenotyping of a transgenic *Caenorhabditis elegans* expressing neuronal amyloid-beta. J. Alzheimers Dis.

[b16] Drake J, Link CD, Butterfield DA (2003). Oxidative stress precedes fibrillar deposition of Alzheimer's disease amyloid beta-peptide (1-42) in a transgenic *Caenorhabditis elegans* model. Neurobiol. Aging.

[b17] Gems D, Riddle DL (2000). Defining wild-type life span in *Caenorhabditis elegans*. J. Gerontol. A Biol. Sci. Med. Sci.

[b18] Gitler AD, Chesi A, Geddie ML, Strathearn KE, Hamamichi S, Hill KJ, Caldwell KA, Caldwell GA, Cooper AA, Rochet JC, Lindquist S (2009). Alpha-synuclein is part of a diverse and highly conserved interaction network that includes PARK9 and manganese toxicity. Nat. Genet.

[b19] Guo N, Peng Z (2013). MG132, a proteasome inhibitor, induces apoptosis in tumor cells. Asia Pac. J. Clin. Oncol.

[b20] Guthrie CR, Greenup L, Leverenz JB, Kraemer BC (2011). MSUT2 is a determinant of susceptibility to tau neurotoxicity. Hum. Mol. Genet.

[b21] Hallengren J, Chen PC, Wilson SM (2013). Neuronal ubiquitin homeostasis. Cell Biochem. Biophys.

[b22] van Ham TJ, Holmberg MA, van der Goot AT, Teuling E, Garcia-Arencibia M, Kim HE, Du D, Thijssen KL, Wiersma M, Burggraaff R, van Bergeijk P, van Rheenen J, Jerre van Veluw G, Hofstra RM, Rubinsztein DC, Nollen EA (2010). Identification of MOAG-4/SERF as a regulator of age-related proteotoxicity. Cell.

[b23] Holmberg CI, Staniszewski KE, Mensah KN, Matouschek A, Morimoto RI (2004). Inefficient degradation of truncated polyglutamine proteins by the proteasome. EMBO J.

[b24] Ittner LM, Ke YD, Delerue F, Bi M, Gladbach A, van Eersel EJ, Wolfing H, Chieng BC, Christie MJ, Napier IA, Eckert A, Staufenbiel M, Hardeman E, Gotz J (2010). Dendritic function of tau mediates amyloid-beta toxicity in Alzheimer's disease mouse models. Cell.

[b25] Jia K, Hart AC, Levine B (2007). Autophagy genes protect against disease caused by polyglutamine expansion proteins in *Caenorhabditis elegans*. Autophagy.

[b26] Kamath RS, Ahringer J (2003). Genome-wide RNAi screening in *Caenorhabditis elegans*. Methods.

[b27] Kikis EA, Gidalevitz T, Morimoto RI (2010). Protein homeostasis in models of aging and age-related conformational disease. Adv. Exp. Med. Biol.

[b28] Kiraga J, Mackiewicz P, Mackiewicz D, Kowalczuk M, Biecek P, Polak N, Smolarczyk K, Dudek MR, Cebrat S (2007). The relationships between the isoelectric point and: length of proteins, taxonomy and ecology of organisms. BMC Genom.

[b29] Lesage S, Brice A (2009). Parkinson's disease: from monogenic forms to genetic susceptibility factors. Hum. Mol. Genet.

[b30] Liachko NF, Guthrie CR, Kraemer BC (2010). Phosphorylation promotes neurotoxicity in a *Caenorhabditis elegans* model of TDP-43 proteinopathy. J. Neurosci.

[b31] Miller J, Arrasate M, Shaby BA, Mitra S, Masliah E, Finkbeiner S (2010). Quantitative relationships between huntingtin levels, polyglutamine length, inclusion body formation, and neuronal death provide novel insight into huntington's disease molecular pathogenesis. J. Neurosci.

[b32] Min K, Kang JS, Lee JH (2010). Modified feeding RNAi method for simultaneous knock-down of more than one gene in *Caenorhabditis elegans*. Biotechniques.

[b33] Morley JF, Brignull HR, Weyers JJ, Morimoto RI (2002). The threshold for polyglutamine-expansion protein aggregation and cellular toxicity is dynamic and influenced by aging in *Caenorhabditis elegans*. Proc. Natl Acad. Sci. USA.

[b34] Nucifora LG, Burke KA, Feng X, Arbez N, Zhu S, Miller J, Yang G, Ratovitski T, Delannoy M, Muchowski PJ, Finkbeiner S, Legleiter J, Ross CA, Poirier MA (2012). Identification of novel potentially toxic oligomers formed in vitro from mammalian-derived expanded huntingtin exon-1 protein. J. Biol. Chem.

[b35] Panowski SH, Wolff S, Aguilaniu H, Durieux J, Dillin A (2007). PHA-4/Foxa mediates diet-restriction-induced longevity of *C. elegans*. Nature.

[b36] Ratovitski T, Chighladze E, Arbez N, Boronina T, Herbrich S, Cole RN, Ross CA (2012). Huntingtin protein interactions altered by polyglutamine expansion as determined by quantitative proteomic analysis. Cell Cycle.

[b1000] Schlessinger A, Punta M, Yachdav G, Kajan L, Rost B (2009). Improved disorder prediction by combination of orthogonal approaches. PLoS ONE.

[b37] Sharad L, Farmer R, Singh A, Jaiswal Y, Wadhwa G (2014). 3D structure generation, virtual screening and docking of human Ras-associated binding (Rab3A) protein involved in tumourigenesis. Mol. Biol. Rep.

[b38] Singam ER, Rajapandian V, Subramanian V (2013). Molecular dynamics simulation study on the interaction of collagen like peptides with gelatinase-A (MMP-2). Biopolymers.

[b39] Skibinski GA, Boyd L (2012). Ubiquitination is involved in secondary growth, not initial formation of polyglutamine protein aggregates in *C. elegans*. BMC Cell Biol.

[b40] Van Raamsdonk JM, Hekimi S (2011). FUdR causes a twofold increase in the lifespan of the mitochondrial mutant *gas-1*. Mech. Ageing Dev.

[b41] Vitalis A, Wang X, Pappu RV (2008). Atomistic simulations of the effects of polyglutamine chain length and solvent quality on conformational equilibria and spontaneous homodimerization. J. Mol. Biol.

[b42] Wright CF, Teichmann SA, Clarke J, Dobson CM (2005). The importance of sequence diversity in the aggregation and evolution of proteins. Nature.

[b43] Youmans KL, Tai LM, Kanekiyo T, Stine WB, Michon SC, Nwabuisi-Heath E, Manelli AM, Fu Y, Riordan S, Eimer WA, Binder L, Bu G, Yu C, Hartley DM, Ladu MJ (2012). Intraneuronal Abeta detection in 5xFAD mice by a new Abeta-specific antibody. Mol. Neurodegener.

[b44] Zhong W, Sternberg PW (2006). Genome-wide prediction of *C. elegans* genetic interactions. Science.

[b45] Zybailov B, Sun Q, van Wijk KJ (2009). Workflow for large scale detection and validation of peptide modifications by RPLC-LTQ-Orbitrap: application to the *Arabidopsis thaliana* leaf proteome and an online modified peptide library. Anal. Chem.

