# Supplementary figures and images for: Proteins in aggregates functionally impact multiple neurodegenerative disease models by forming proteasome-blocking complexes
Source: Aging Cell. 2014 Dec 16;14(1):35–48. doi: 10.1111/acel.12296 (PMC4326912; doi:10.1111/acel.12296)

Supporting Figure 1

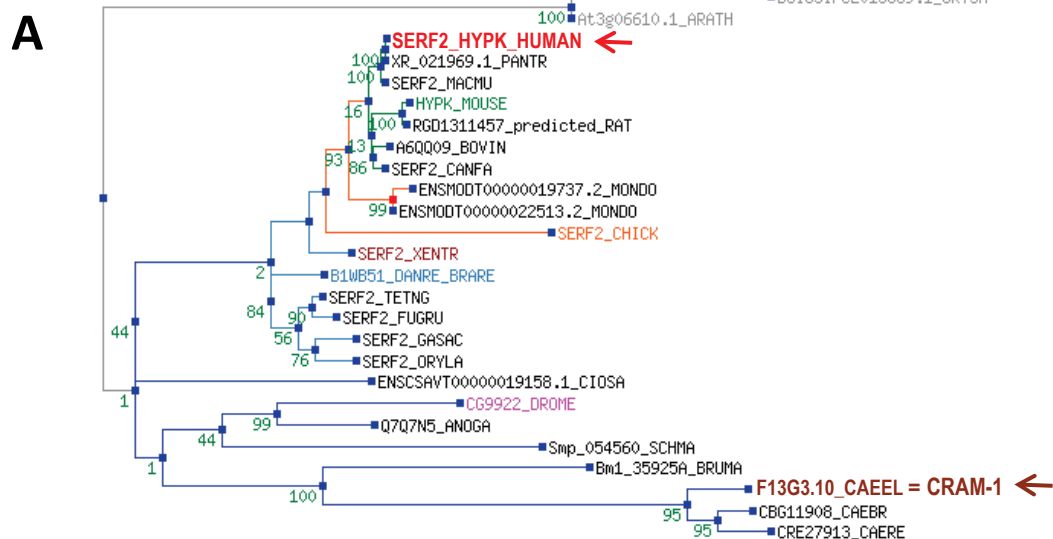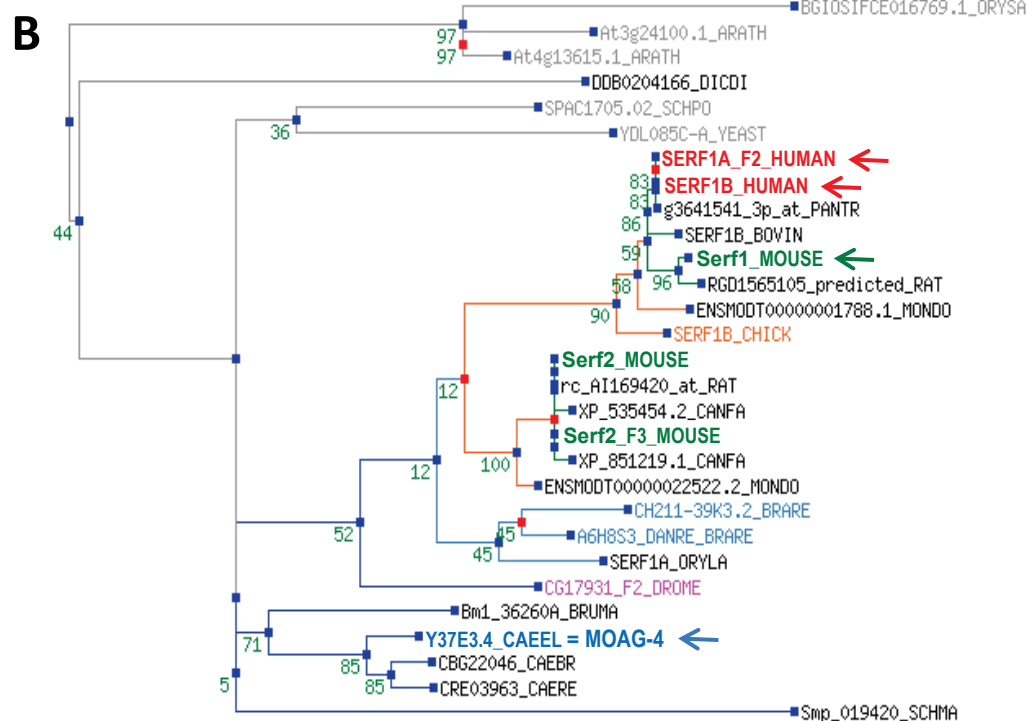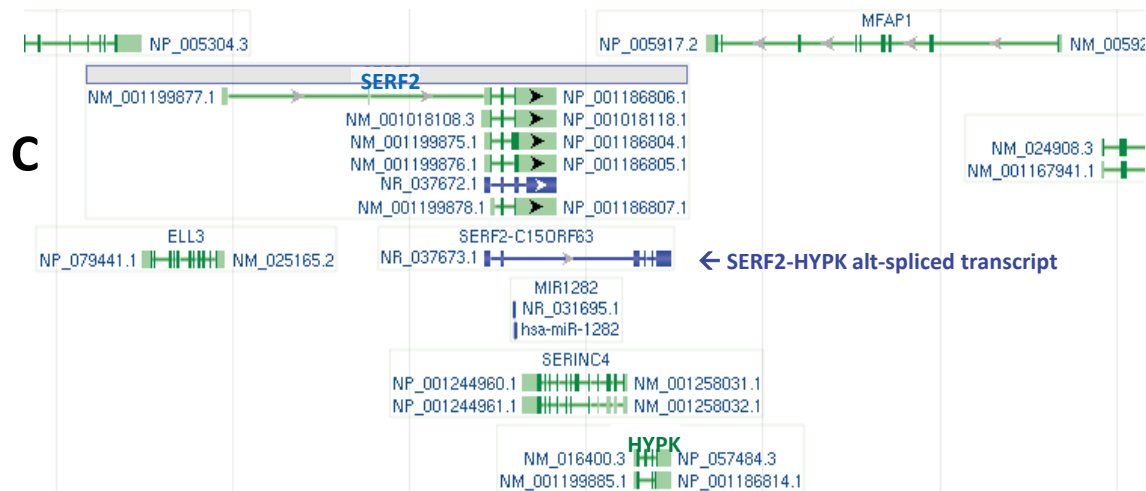

Supplement: Supplementary file 1 [file acel0014-0035-sd1.pdf]
