# Supplementary material for: Proteins in aggregates functionally impact multiple neurodegenerative disease models by forming proteasome-blocking complexes
Source: Aging Cell. 2014 Dec 16;14(1):35–48. doi: 10.1111/acel.12296 (PMC4326912; doi:10.1111/acel.12296)
Supplement: Supplementary file 2 [file acel0014-0035-sd2.docx]

**Supporting Figure 1** SERF2-HYPK, a partially duplicated gene, is the closest human ortholog of CRAM-1. (**A**) Orthology tree ([www.treefam.org](http://www.treefam.org)), for F13G3.10 (CRAM-1), shown in compact form. (**B**) Orthology tree for MOAG-4, as in “A”. (**C**) Human SERF2 and HYPK are adjacent, cotranscribed genes (map generated by WormBase, [www.wormbase.org](http://www.wormbase.org)). The mRNA indicated as “SERF 2-HYPK alt-spliced transcript” implies that SERF 2 and HYPK can be cotranscribed.

**Supporting Table 1. Proteins identified with >95% confidence in Q40::YFP aggregates**

| **Day 4, Sarcosyl-soluble** | **Day 4, Sarcosyl-insoluble** | **Day 7, Sarcosyl-soluble** | **Day 7, Sarcosyl-insoluble** |
| --- | --- | --- | --- |
| **PQN-53**, protein with a prion-like Q/N-rich domain | **PQN-53**, protein with a prion-like Q/N-rich domain | **UNC-108**, a small Rab GTPase needed by neurons for locomotion | **PQN-53**, protein with a prion-like Q/N-rich domain |
| **DNJ-11**, DnaJ/Myb domain ortholog, human ribosome -associated chaperones | GFP (YFP), encoded by a Q40::YFP transgene | KLP-15, a kinesin-like protein, homologous to human KIFC3 | GFP (YFP), encoded by a Q40::YFP transgene |
| **HIS-1**, an H4 histone closely homolgous to human H4 | C48E7.6, orthologous to human chondroitin sulfate proteoglycan 4 | **HIS-1**, an H4 histone closely homolgous to human H4 | LET-2, an alpha-2 type-IV collagen, part of the M/E basement membrane |
| NLP-29, an antimicrobial, neuropeptide-like protein | F59E12.9, an unknown protein | **NLP-31** comprises 5 antifungal peptides | **NLP-31** comprises 5 antifungal peptides |
| SKP-1, ortholog of human SKI-binding protein SKIP | Y116A8C.465, unknown protein | RPL-29, large ribosomal protein subunit (L29) | **ATX-2**, homolog of human Ataxin-2-like protein |
| T23G5.2, highly similar to human SEC14-like prot. 1 | F42G8.8, homolog of human Ser/Thr protein phosphatase PP1-γ c.s. | RSR-2, ortholog of human ser/arg-repetitive matrix protein 1 | CPSF-2, orthol. to human cleavage & polyadenyl’n specificity factor subunit 2 |
| ZK1248.17, unknown protein | **GLT-5**, ortholog of excita­tory AA-transporter 2 | **GLO-4**, guanine nucleo­tide exchange factor | **UNC-2**, Ca-channel α s.u. DOPA, serotonin neurons |
|  | F32D8.15, TM hom­olog of a TRP channel subunit | OCR-3, TM homolog of a TRP channel protein | **PQN-22** protein with prion -like Q/N-rich domain |
|  | ZK285.2, unknown protein | FIPR-23, fungus-induced protein related | CEH-1, homeobox-containing protein |
|  | F40D4.13, unknown protein, RNAi lowers fat | F55H12.3, unknown protein | Y39E4B.10, unknown protein |
|  |  | Y119C1B.3, unknown protein | K05C4.3, unknown protein |
|  |  | **WEE-1.3**, Wee1 family cdc2-inhibiting kinase | C03C11.1, unknown protein |
|  |  | ZK1236.1, conserved ortholog of human mitoc. translation factor GUF1 | ZBP-1 homolog of human IGF-2 mRNA-BP2 protein; prion-like Q/N-rich domain |
|  |  | **WHT-6**, ABC transporter | MRP-8, ABC transporter, (=MDR-assoc. protein 1) |
|  |  | C31B8.1, unknown protein | **RSKN-1** conserved ortho- log of human S6 kinase α1 |
|  |  |  | **RGS-2**, GTPase activating regulator of G-prot.signals |
|  |  |  | W04G3.7, unknown prot. |
|  |  |  | SMC-6, HR/DNA-repair, ortholog of human SMC6 |
|  |  |  | F39C12.1, unknown prot. |
|  |  |  | F13G3.10 (**CRAM-1**), weakly homol. to HYPK |
|  |  |  | T22H2.4, unknown prot. |

Proteins in **bold** were tested for effect of RNAi targeting the encoding gene in AM141 (e.g., Supporting Table 2). **Supporting Table 2.**

**Aggregate count is reduced by RNAi targeting proteins that co-aggregate with Q40::YFP**

|  |  |  | | | | | | |  | |
| --- | --- | --- | --- | --- | --- | --- | --- | --- | --- | --- |
| **RNAi** | | | **Aggregate count per worm** ±**SEM** | **Normalized aggregates per worm** | | | **Norm. SEM** | | ***P*, 1-tailed**  ***t* test vs. FV** | |
| **None (FV)** | | | 68.9 ± 1.7 | | 1.00 | 0.025 | | ‒ | |  |
| ***cram-1*** | | | 37.2 ± 2.3 | | 0.54 | 0.033 | | <0.005 | |  |
| ***pqn-22*** | | | 45.3 ± 0.9 | | 0.66 | 0.021 | | <0.001 | |  |
| ***pqn-53*** | | | 59.0 ± 1.1 | | 0.86 | 0.019 | | <0.002 | |  |
| ***atx-2*** | | | 60.9 ± 1.6 | | 0.88 | 0.027 | | <0.005 | |  |
| ***unc-108 (rab2A homolog)*** | | | 56.3 ± 2.0 | | 0.82 | 0.036 | | <0.001 | |  |

RNAi treatment of strain AM141 (expressing *unc-54p*/Q40::YFP in muscle) began at the L4/adult moult, to avoid possible developmental effects of knockdown. Images were acquired for 8‒16 worms at day 5 posthatch, and quantified using DotCount software (<http://reuter.mit.edu/software/dotcount>). Additional genes were targeted in other experiments (see Supporting Table 1), and each gene was tested at least twice.

**Supporting Table 3.**

**Oleuropein treatment is additive with *cram-1* RNAi in suppressing Q40::YFP aggregation**

|  |  |  | | | | | | |  | |
| --- | --- | --- | --- | --- | --- | --- | --- | --- | --- | --- |
| **Treatment** | | | **Aggregate count per worm** ±**SEM** | **Normalized aggregates per worm** | | | **Norm. SEM** | | ***P*, 1-tailed**  ***t* test vs. FV** | |
| FV control | | | 62.8 ± 1.0 | | 1.00 | 0.016 | | ‒ | |  |
| *cram-1* RNAi | | | 46.4 ± 1.3 | | 0.74 | 0.028 | | <8E‒12 | |  |
| FV, DMSO control | | | 60.5 ± 0.9 | | 0.96 | 0.014 | | N.S. | |  |
| DMSO (OP50) control | | | 59.2 ± 0.9 | | 0.94 | 0.016 | | N.S. | |  |
| oleuropein (80 µg/ml)/OP50 | | | 48.3 ± 1.4 | | 0.77 | 0.028 | | <3E‒7 | |  |
| *cram-1* RNAi + oleuropein | | | 37.8 ± 1.6 | | 0.60 | 0.042 | | <2E‒13 | |  |

All treatments of strain AM141 began at hatch. Images were acquired for 14‒21 worms early on day 5 posthatch, and quantified using DotCount (see Supporting Table 2). The effect of *cram-1* RNAi plus oleuropein, 0.60, approximately equals the product of the effects of *cram-1* RNAi vs. FV control (0.74) and oleuropein alone relative to its control (0.77/0.94=0.82): 0.74 x 0.82 = 0.61.
